# Host Genetics and Gut Microbiome: Perspectives for Multiple Sclerosis

**DOI:** 10.3390/genes12081181

**Published:** 2021-07-29

**Authors:** Alessandro Maglione, Miriam Zuccalà, Martina Tosi, Marinella Clerico, Simona Rolla

**Affiliations:** 1 Department of Clinical and Biological Sciences, University of Torino, 10100 Torino, Italy; alessandro.maglione@unito.it (A.M.); marinella.clerico@unito.it (M.C.); 2 Department of Health Sciences, Center on Autoimmune and Allergic Diseases (CAAD), Università del Piemonte Orientale, 28100 Novara, Italy; miriam.zuccala@med.uniupo.it (M.Z.); martina.tosi@uniupo.it (M.T.)

**Keywords:** Multiple Sclerosis, gut microbiome, host genetics, dysbiosis

## Abstract

As a complex disease, Multiple Sclerosis (MS)’s etiology is determined by both genetic and environmental factors. In the last decade, the gut microbiome has emerged as an important environmental factor, but its interaction with host genetics is still unknown. In this review, we focus on these dual aspects of MS pathogenesis: we describe the current knowledge on genetic factors related to MS, based on genome-wide association studies, and then illustrate the interactions between the immune system, gut microbiome and central nervous system in MS, summarizing the evidence available from Experimental Autoimmune Encephalomyelitis mouse models and studies in patients. Finally, as the understanding of influence of host genetics on the gut microbiome composition in MS is in its infancy, we explore this issue based on the evidence currently available from other autoimmune diseases that share with MS the interplay of genetic with environmental factors (Inflammatory Bowel Disease, Rheumatoid Arthritis and Systemic Lupus Erythematosus), and discuss avenues for future research.

## 1. Introduction

Multiple Sclerosis (MS) is a chronic inflammatory demyelinating disease that causes demyelination of oligodendrocytes in the Central Nervous System (CNS) [1]. MS is today the leading cause of non-traumatic disabilities in young adults in Europe [2] with a prevalence of over 2 million cases worldwide [3]. Epidemiology studies have shown a sex difference in the prevalence and progression of the disease: MS has a 2:1~3:1 female to male ratio [4]. The relapsing form of MS is more frequent in young women [5] while in men MS shows higher incidence at older ages and a more severe progressive course [6].

The current definition of the immunopathogenesis of MS describes the initial activation of self-reactive T and B lymphocytes in the peripheral lymph nodes and their differentiation into effector cells. T lymphocytes, in particular CD4 + T cells, are the most involved in MS pathogenesis [7,8]. Pro-inflammatory T helper cells (Th) 1, Th 17 and Th 22 are activated in the periphery following a deficit of regulatory T lymphocytes (T reg), which lose the ability to suppress the autoreactive response [9]. Remarkably, MS patients show an increased count of Th17 cells in the peripheral blood [7], cerebrospinal fluid and perivascular space in the CNS [8,10,11,12], following an invasion of the CNS through the blood-brain barrier (BBB). The disruption of the BBB is recognized as an important early feature of MS pathogenesis. T and B cells cross the BBB, which is discontinuous, and access the CNS where antigen presenting cells (APC) reactivate them. In the CNS, the activated immune system reacts against myelin components. Inflammation is supported by the production of cytokines and chemokines and by the recruitment of other self-reactive cells from the peripheral blood. In particular, activated B cells mature into plasma cells and produce antibodies, maintaining and reactivating CD4 + T cells that produce proinflammatory cytokines. In the later stages of the disease, the inflammatory response is supported by the activation and polarization of microglia, leading to chronic neurodegeneration [1,13,14].

To date, the etiology of MS remains largely unknown, but it is considered a multifactorial disease in which both genetic and environmental factors could influence the immune system development and response in individuals, predisposing them to MS onset [3,4]. 

Although genetics account for ∼30% of disease susceptibility and first-degree relatives of patients show susceptibility for MS [15,16], MS is not an inheritable disease. Susceptibility and protective alleles have been identified in the Region of the Major Histocompatibility Complex (HLA, Human Leukocyte Antigens) and in several genes related to the immune response [17,18]. Among environmental factors, the most recognized as linked to the risk of developing MS disease are Epstein–Barr virus (EBV) infection [19,20], smoking habitude [21] and vitamin D deficiency [22]. Recently, great attention has been paid to gut microbiome composition [23] and several studies have tried to identify which microorganisms may promote or counter its pathogenesis and progression [24,25]. Interactions between environmental factors and genes are considered to determine the remaining 70% of disease risk. How these complex networks of interactions ultimately drive disease susceptibility remains to be elucidated.

## 2. Genetic Factors in MS Patients

It is known that the rate of MS recurrence in families is 20%, and concordance between monozygotic twins is 24–30% while between dizygotic twins is only 3–5%, which is comparable to that of normal brothers [26,27]. Before the era of genome-wide association studies (GWAS) the only genetic factor implicated in MS susceptibility was on the HLA region on the short arm of chromosome 6 that extends for about 4 Mb. In particular, a strong association was observed with the DRB1*15.01 allele, which confers a threefold increase in risk [28]. Further studies identified other associations with different HLA loci independently of the DRB1*1501: the allele HLA-DRB1*0301 and the allele HLA-DRB1*1303, which confer an MS risk, and the protective allele HLA-A*0201 and a variant that tags HLA-DPB1*0301 [29]. In the last ten years, international GWAS studies analyzing large datasets have identified 200 loci involved in susceptibility to the disease separate from the HLA region. This discovery was mainly due to the contribution of three international studies in 2011 [18], 2013 [30], and 2019 [31]. In particular, with the latest GWAS study published in 2019 [31], in which 39,000 individuals from nine different populations were tested and 331,536 Single Nucleotide Polymorphisms (SNPs) analyzed, the number of statistically independent associations with MS susceptibility have been increased to 233. Of these, 32 were within the HLA region, one in chromosome X, and 200 in the autosomal non-HLA region. The genome-wide and suggestive effects jointly could explain about 48% of the estimated heritability. This study confirmed the enrichment for MS susceptibility loci in many different immune cell types and tissues, but on the other hand, it did not find enrichment in tissue-level CNS profiles. Analyzing data generated from human induced Pluripotent Stem Cells (iPSC)-derived neurons as well as from purified primary human astrocytes and microglia, the authors observed that enrichment for MS genes is seen in human microglia but not in astrocytes or neurons. Despite these efforts in identifying these associated variants and loci, and although several signals are near genes involved in immunologic processes, the effector mechanisms for most associations remain unknown.

Until now, very few fine mapping analyses have been conducted to identify a primary causal variant or gene. Among these, we reported the case of the Interleukin-7 Receptor (IL7R) gene (rs6897932) [32,33], the case of the Tumor Necrosis Factor (TNF) receptor superfamily member 1A (TNFRSF1A) gene (rs1800693) [34] and the case of the TNF Superfamily Member 13B (TNFSF13B) locus (BAFF-var, insertion-deletion) [35]. More recently, a study published by the International Multiple Sclerosis Genetics Consortium attempted to examine in depth the role of low-frequency and rare variants in susceptibility to MS. This study analyzed 32,367 MS cases and 36,012 controls in detail though an array platform called Exome chip (Illumina technology), which contains almost 200,000 SNPs (rare synonymous and non-synonymous SNPs and common synonymous SNPs) mapping in coding regions enriched in rare variants (MAF < 0.01) [36]. They found a significant association for seven low-frequency variants in six genes outside the HLA region. Two of these variants were present in genes identified by MS GWAS and showed linkage disequilibrium with the common variant previously reported in GWAS [18], while the remaining signals were novel and did not show linkage disequilibrium with common variant association signals found through GWAS. The identified genes showed a clear immunological function, particularly in T cell development. This work demonstrated that nearly 5% of the heritability of MS can be explained by the already identified coding low-frequency variants and that more low and rare frequency variant associations remain to be discovered using larger sample sizes to increase statistical power.

The remaining fraction of the risk commonly known as “missing heritability” is likely due to still unknown common variants characterized by much smaller effects, below the detection limits of the GWAS studies conducted so far. Some authors have proposed that a substantial portion of the missing heritability lies in genetic interactions between known variants, the so-called phantom heritability [37]. Similarly, gene–environment interactions, cis/trans-regulators of allelic expression, unidentified rare and penetrant semi-private variants, population and/or disease heterogeneity, the neglect of the analysis of sex chromosomes, and hidden epigenetic effects may all contribute to the missing heritability. 

## 3. The Gut Microbiome and MS

### 3.1. The Human Gut Microbiota

All microorganisms, including bacteria, archaea, fungi, and viruses, exist in an ecosystem called a microbiota, and the collective genomic, protein, and metabolite content of all the microbes in this given ecosystem is called a microbiome [38]. The human microbiota colonizes virtually every surface of the human body exposed to the external environment: skin, urogenital tract, respiratory tract, and digestive tract, but more than 95% of the microbiota is located in the large intestine. Here, about 10^14^ different populations of microorganisms live, with a number of genes at least 100 times larger than the number of human genes in the body [39]. This large number of intestinal microbes has evolved over a long period of time, becoming an inseparable part of the host and playing an important role in maintaining the health of the body. 

The relative composition of the gut microbiota can be studied by collecting a stool sample. After DNA extraction, a following metagenomic analysis is conducted through two main techniques: analysis of the 16S subunit ribosomal RNA (only for bacteria), or whole genome sequencing [40,41]. The sequences obtained are not directly identified by species or phyla, but are classified into Operational Taxonomic Units (OTUs), defined as groups of microorganisms united by a DNA similarity of at least 97% [41]. The sequences obtained are mapped onto reference genomes to obtain the relative quantification of taxa, from which the microbial diversity is derived, or subjected to de novo assembly for the identification of new phyla [41]. The Metagenomics of the Human Intestinal Tract and the Human Microbiome Project have provided the most integrated view of human-associated microbes: 2172 species have been isolated in human gut microbiota [42], and a healthy gut microbiota is mainly dominated by Bacteroidetes and Firmicutes, while Proteobacteria, Verrucomicrobia, Actinobacteria and several other phyla are present in smaller proportions [43]. 

Gut microbiota and host develop an intimate mutualistic relationship. Normal gut microbiota is involved in specific functions in host nutrient metabolism: it derives its nutrients from host dietary components and produces source of energy for the host; many taxa are able to synthetize short chain fatty acids (SCFA) such as butyrate, propionate and acetate from fermentation of carbohydrates that escaped proximal digestion and indigestible oligosaccharides [44]; it contributes to lipid and protein metabolism, by suppressing the inhibition of lipoprotein lipase activity in adipocytes, and through microbial proteinases and peptidases acting in tandem with human proteinases [45]; it is also involved in synthesis of vitamin K and several components of vitamin B. Gut microbiota functions also include xenobiotic and drug metabolism, maintenance of structural integrity of the gut mucosal barrier, immunomodulation, and protection against pathogens.

### 3.2. Factors Influencing the Composition of Gut Microbiota 

Changes in the composition of the gut microbiota accompany the individual from infancy, through adolescence and adulthood, to old age [46]. The first traces of microbial colonization have been identified in utero, amniotic fluid and placenta although this remains controversial [47,48,49]. However, the large-scale colonization of the human intestine by microbes begins at birth [48,50]. The first differences in microbial composition are observed between caesarean and natural delivery. Natural childbirth exposes the newborn to vaginal bacteria, mainly Lactobacillus, while Caesarean section favors contact with the skin, where Actinobacteria mainly reside [40,48,50]. Further changes are introduced by the type of feeding (for example breastfeeding or formula feeding and then weaning) [40]. After the first year of age, an infant microbiota resembles that of a young adult and stabilizes, but its composition is continuously influenced by endogenous and exogenous factors such as genetics, sex, diet and drugs. [40,51]. Finally, aging directly acts on the composition of the microbiota with a decrease in microbial diversity and a decrease in Bifidobacteria and Firmicutes of the genus Clostridium [50].

Microbial turnover in the adult gut is subject to natural pressures by the host. The epithelium acts as a physical barrier between microbes and the host’s body by sensing microbes and mediating the activation of a proper mucosal immune response (innate and adaptive). It can suppress pathogens using a so-called “punitive mechanism”. Alternatively, the host can promote proliferation of beneficial microbes through a “positive control”: by feeding the preferred species via epithelial-derived nutrients or by using secretion factors that can influence the adhesion of microbial cells to intestinal mucus (e.g., mucin glycan residues and IgA proteins) [52].

Host gene composition and bacterial gene composition establish an obvious mutual relationship in which metabolites and molecules interact, maintaining a balance between the gut microbiota and the environment, as discussed in depth later. Bacteria can synthesize metabolites on the basis of genetic composition (i.e., lipopolysaccharide (LPS), SCFAs and amyloids) or regulate host gene expression by using miRNAs (which silence the expression of target genes). Conversely, fecal miRNAs can regulate bacterial composition by specifically targeting bacterial genes [53]. 

Sex hormones and gut microbiota influence each other and parallel metabolic and immune changes through the lifespan [54]; interestingly, adoptive transferred male microbiota to recipient females resulted in elevated testosterone and metabolomic changes and delayed the onset and severity of type 1 diabetes in a mouse model, suggesting that the higher female risk for autoimmunity can be influenced by sex differences in the gut microbiome [55]. 

Dietary habits and food types can influence microbial composition as well. The effect of diet on gut microbiota has been investigated at several levels, from specific nutrients to diet styles [56]. 

Antibiotics can strongly affect the components of gut microbiota: adult gut microbiomes seem not to be resilient to repeated antibiotic administration; furthermore, antibiotics taken early in life could deeply impact the gut microbiome composition, resulting in predisposition to obesity, asthma, inflammatory bowel disease and other disorders such as autoimmunity [57]. 

### 3.3. Gut Microbiota-Immune-Brain Interactions

An increasing number of studies have suggested that gut microbiota and CNS communicate constantly during life stages, connecting the emotional and cognitive centers of the brain with peripheral intestinal functions [46,58,59] via the microbiome–gut–brain axis that consists of molecular pathways, belonging to the endocrine, immune, nervous, and metabolic systems, interacting with each other [46,60,61,62]. The gut microbiota interacts with the CNS by producing various metabolites ranging from sugars and SCFA to neurotransmitters including serotonin, γ-aminobutyric acid (GABA), norepinephrine and dopamine [63]. Furthermore, it stimulates the peripheral immune system by inducing the release of cytokines and chemokines which, in addition to locally regulating bacterial concentrations, can infiltrate the blood and lymphatic system and therefore have effects on the CNS [63]. Moreover, it can interact with the CNS by stimulating the vagus nerve [63]. Conversely, biochemical changes in the central nervous system can alter the microbial composition and immune cell responses in the gut microbiome through the hypothalamic–pituitary–adrenal (HPA) axis [63].

Interactions between the intestinal microbiota, the peripheral immune system and the CNS are essential for the life of the host. Studies in germ-free (GF) rodents have shown the crucial role of the microbiota in key aspects of neurodevelopment, neuroinflammation and behavior, reviewed in detail in [46].

Gut associated lymphoid tissue (GALT) is composed of cells with innate and adaptive immunity. The cells with innate immunity distinguish between potentially pathogenic microbial components and harmless antigens by means of “pattern recognition receptors” (PRRs), which allow mammalian cells to recognize characteristic conserved molecules present on microorganisms and lead to activation of Th17, Treg and IgA-producing B cells with adaptive immunity [64]. Mucous IgA secreted through the epithelium coats and agglutinates their targets to prevent their direct interaction with the host. This avoids the potentially harmful stimulation of the immune system in the mucous membranes by the lumen contents and also preserves the regular composition of the microbiota [65]. 

In the lamina, 30/40% of the memory CD4+ T lymphocytes are Th17 cells. Under physiological conditions, their typical cytokines, interleukin (IL)-17A, IL-17F and IL-22, stimulate the production of antimicrobial proteins by intestinal epithelial cells and the formation of tight junctions between these cells [66]; they also mediate IgA transport [67] and neutrophil recruitment [68]. Consequently, Th17 cells play an indispensable role in preventing infection by various species of pathogenic bacteria and fungi. Since Th17 cells can induce harmful inflammation, their response in the gut is strictly regulated by induction of Treg cells. Intestinal Treg cells comprise both CD4(+) T cells that express Foxp3 and Tr1-like cells that produce IL-10 and can be generated in conditions that also promote effector T cell responses; both play an important functional role in promoting tolerance to food antigens and gut microbiota [69], as well as in suppressing tissue damage caused by immune responses against pathogenic bacteria [70].

Specific bacteria preferentially induce specific CD4+ T subtypes. For example, segmented filamentous bacteria (SFB) induce the differentiation of Th17 cells in the ileum, while strains of *Clostridia* and *Bacteroides fragile* favor the differentiation of Tregs in the colon. [71]. Gut-induced Th17 cells can became pathogenic under specific stimuli, such as high IL-23 production, salt, or long chain fatty acids. They can migrate to the draining lymph nodes of target organs, such as the CNS, contributing to autoimmune disease through cross-reactivity between microbial peptides and self-antigens (model of molecular mimicry). Alternatively, microbiota-specific Th17 cells migrate towards the lymph nodes and lower the activation threshold of self-reactive effector T cells (T cell threshold model) [71], inducing a harmful immune response to self-antigens. 

### 3.4. Evidences from Experimental Autoimmune Encephalomyelitis (EAE)

The first evidence of the involvement of gut microbiota in MS comes from experiments on the EAE, the most commonly used model for MS, which resembles the inflammation and demyelination processes of the human counterpart. Specific components of gut microbiota have the capability to promote or prevent EAE. A first study by Berer et al. [72] showed that a germ-free environment has been associated with a milder EAE disease course. This result was not surprising as germ-free mice show extreme defects in immune system development, but clearly demonstrates the essential role of commensal-mediated immune system development in this disease. GF EAE mice have lower levels of the proinflammatory cytokines IFN-γ and IL-17A, both in the gut and the CNS, and display an increase in Treg cells [73]. Of further note, gut microbiota influences blood-brain barrier (BBB) permeability in mice. Germ-free mice have disrupted BBB tight junctions when compared to pathogen-free adult mice. The permeability is overcome by colonization of the germ-free mice with conventional gut flora. This observation suggests that early colonization by microflora may be essential in the development of normal BBB function [74].

Intestinal colonization by segmented filamentous bacteria (SFB), a *Clostridia*-related species that displays features between an obligate and facultative symbiont, promoted the production of IL-17 in the intestine and induced the expansion of Th17 in the CNS [73]. The abundance of SFB, together with gut barrier function, is regulated by the IL-23R/IL-22 pathway. When the intestinal barrier is disrupted, systemic dissemination of microbial products occurs, which invokes the IL-23 pathway and initiates barrier repair, as well as Th17 responses aimed at neutralizing invading commensal microbes. Moreover, SFB-induced-IL-23 results in production of IL-22 that triggers a complex circuit that promotes IL-17 expression in T cells, especially in the terminal ileum, which is the site of SFB attachment to the epithelium. SFB-induced activation may also result in the generation of autoreactive Th17 cells in response to presentation of autoantigens in the setting of a breached intestinal barrier [75].

Conversely, the Gram-negative bacteria *Bacteroides fragilis*, which also reside in the human gut microbiome, promote immune homeostasis. Polysaccharide A (PSA), the most abundant capsular polysaccharide expressed by B. fragilis, protected mice from EAE by inducing conversion of CD4+ T-cells into IL-10-producing Foxp3+Tregs via Toll like receptor (TLR)2 and suppressed Th17 responses [76,77]. Further oral administration of PSA was associated with a lower “clinical” score and was able to stimulate the adaptive immune system towards an anti-inflammatory status [76]. 

Moreover, dietary metabolites may influence systemic autoimmune response. SCFA improved the course of EAE by long-lasting imprinting of gut-derived Treg cells, while long-chain fatty acids (LCFA) exacerbated EAE disease and induced expansion of Th17 cells in the gut [78].

### 3.5. Evidences from Clinical Studies in MS Patients

Studies in MS are still at the early stage of development and limited to case-control examinations of the gut microflora in MS patients versus healthy controls (Table 1). Although changes in some commensal bacteria have been consistently detected in different microbiota studies in MS, the results vary between different studies. These discrepancies may reflect differences in the ethnic, genetic, lifestyle, and dietary backgrounds of the patient cohorts analyzed. However, some peculiarities have been highlighted in the intestinal microbiota of subjects with MS compared to controls.

Decreased abundance of *Clostridia* [79], *Bacteroides* and *Parabacteroides* [79,80,81], *Butyricimonas* [82], *Faecalibacterium* [83], *Prevotella* [79,80,81], *Lactobacillus* [81], *Adlercreutzia* and *Collinsella* [81] has been observed in MS subjects with respect to controls. Conversely, MS subjects showed increased abundance of *Metanobrevibacter* [82], *Acinetobacter calcoaceticus* [80,84], *Akkermansia muciniphila* [78,80,82], *Pseudomonas*, *Mycoplana*, *Blautia* and *Dorea* [81] with respect to controls. According to these observations, some relationships involving the pathogenetic mechanism of MS can be conjectured. Firmicutes and Bacteroitedes are SCFA producers, and SCFA metabolism has been related to MS pathogenesis [24,25] and progression [85]. In particular, *Faecalibacterium* and *Bacteroitedes* are butyrate and propionate producers. A reduced abundance of these bacteria in MS patients can be associated with a decreased inhibition of the immune system inflammatory response through the NF-Kb pathway by SCFAs [24] or with other mechanisms of immunetolerance, including those related to Treg cells [24]. Notably, the polysaccharide A (PSA) of *Bacteroides fragilis* can induce IL-10 dependent enhanced conversion of Treg cells and protect against autoimmunity [24]. An imbalance in *Clostridia* can exert diverse effects. Some clostridial species found in reduced numbers in the gut microbiota of MS patients can induce colon regulatory T cells (Tregs), which prevent autoimmunity and allergies [79]. 

Conversely, MS subjects showed increased immunoreactivity to the Epsilon toxin (ETX) produced by *Clostridium perfringens* [86], indicating prior exposure to ETX. ETX’s tropism for the BBB and binding to oligodendrocytes/myelin makes it a stimulating candidate for nascent lesion formation in MS [86]. *Metanobrevibacters* are methanogenic archaea that can induce dendritic cell activation and pro-inflammatory effects on cells with adaptive immunity [24]. Changes in the abundance of *Akkermansia*, *Acinetobacter* and *Bifidobacterium* have been associated with impaired ability to promote the differentiation of Tregs, or with the ability to promote Th1 cell differentiation in PBMC cultures [80,84]. Higher incidence of EAE after MS fecal transfer in GF mice has been observed [80] but the roles of single taxa remain debated. For example, the effect of *Akkermansia* on MS is controversial: studies from multiple cohorts found an increase of *Akkermansia muciniphila* in MS gut microbiotas [80,84]; by contrast, *Akkermansia* showed anti-inflammatory properties in EAE after fecal transplant from EAE-peak to EAE-susceptible mice, expanding Treg cells by a mi-RNA dependent mechanism [87]. According to these observations it is evident that the number of hypotheses that link specific microorganisms to MS pathogenetic mechanism is constantly growing.

## 4. Interactions between Gut Microbiota and Host Genes

### 4.1. Heritability of Gut Microbiome

Associations between the human genome and microbiome composition have been extensively studied in recent years. Microbiome and host genetics are strictly correlated [88]: the first plays a role in human health and disease by influencing the host’s traits, disease susceptibility, and treatment response; the second influences microbiome composition [89]. In particular, studies on twins demonstrated that the gut microbiomes of monozygotic (MZ) twins are significantly more similar than those of dizygotic (DZ) twins, indicating that host genetic factors are involved in modulating gut microbiome composition [88,90]. The first study by Goodrich et al. [91] was performed on a large cohort of MZ and DZ twin pairs (977 individuals), which allowed them to assess the impact of genotype and early shared environment on gut microbiota. This study was fundamental for the further and larger study on the Twins UK cohort (416 twin pairs). This new study in 2016 demonstrated significant heritability of overall microbiome composition [92]. Several heritable taxa have been identified: the family *Christensenellaceae* has been demonstrated to have the highest heritability; however, the archaea family *Methanobacteriacae*, the phyla *Firmicutes*, *Actinobacteria*, *Tenericutes*, and *Euryarchaeota* were shown to be more heritable, while the highly abundant Bacteroidetes phylum shows very little heritability. In a Canadian cohort, 20 bacterial taxa were found to be heritable and included five taxa reported in the UK cohort [93]. These results indicated that some bacteria are affected by host genetics in the overall human population, whereas variation of the overall composition of the gut microbiome is determined by environmental factors. 

### 4.2. MGWAS Studies

In the last decade many microbiome genome-wide association studies (mGWAS) have been conducted, performing both 16S RNA sequencing and whole-metagenome sequencing to identify hosts’ genetic polymorphisms that interact with the microbiome and influence its architecture and functional diversity [88,89,90,94]. Associations between microbiome and variants located in genes involved in immunity and metabolism have been found from these studies, despite the relatively small sample sizes of healthy subjects (Table 2) [89]. 

The first study by Blekhman et al. [95] focused on 93 individuals and included host information derived from Human Microbiome Project. Eighty-three associations were identified between human loci and the microbiome using the shotgun metagenomic sequencing method, but only one association, between the lactase gene *LCT*, which encodes lactase-phlorizin hydrolase (LPH) and the abundance of *Bifidobacterium* (from the phyla *Actinobacteria*), has been validated in other cohorts. The *LCT* gene is an example of how dietary changes affect the relationship between genetics and microbiome. SNPs at the *LCT* locus that promote continued LPH production enable lactose metabolism in adults, whereas *LCT* variants associated with reduced LPH activity are implied with lactose intolerance and can promote the growth of lactose-fermenting bacteria in the colon, but only if the individual consumes dairy products [90]. In the same year, a second mGWAS was conducted by Davenport et al. using 16S rRNA sequencing on a larger cohort of 127 individuals living in North America. Results of the study demonstrated a correlation between SNPs in regions of the phospholipase D1 *PLD1* gene and abundance of the genus *Akkermansia* [89,90,94,95,96]. In 2016 a major study was conducted by Wang et al. [97] using 16S rRNA to analyse microbiomes of more than 1800 healthy European subjects. By applying an analysis of variance (ANOVA) approach, the authors identified 42 host genetic loci that affected *β diversity*, a measure of variability in gut microbiome composition between different environments. Individually, each locus explained only a small proportion of the total variability between different hosts, but together the loci explained almost 10% of the inter-individual gut microbiome variability. In particular, it was shown that variants in one of the identified loci, the Vitamin D Receptor (*VDR)* gene, were associated with microbiome composition [97]. In the same study, by applying an alternative statistical method, 40 other loci were found to be correlated with bacterial taxa and some genes were shown to affect the composition of the gut microbiome, such as *LCT*, Fucosyltransferase 2 (*FUT2*) and Nucleotide Binding Oligomerization Domain Containing 2 (*NOD2*), although these associations did not reach genome-wide significance [97]. Moreover, Turpin’s group performed a family case study, applying 16S RNA sequencing to Canadian Caucasian asymptomatic first-degree relatives of individuals with Crohn’s disease and to a replication cohort including 270 individuals from 123 families [90,93]. They identified 20 taxa as being heritable: seven new and 13 found in a previous twin study [97]. This information was confirmed subsequently in a twin study using a different approach: whole-metagenome sequencing. The same method was followed in 2016 by Bonder et al. [98], whose study included more than 1500 healthy individuals in three different Dutch cohorts. The authors confirmed the correlation between the *LCT* gene and *Bifidobacterium* abundance and identified nine other human loci associated with bacterial taxa, as well as 33 loci associated with bacterial functional pathways using a conventional P value threshold for GWAS (*p* < 5 × 10^−8^) [94]. One of the most recent studies [99] tried to replicate Blekhman’s results from the 2015 experiments: they considered the same cohort, but used more stringent thresholds. As a result, their untargeted GWAS could not identify any significant associations, whereas the original study identified 83 associations [89]. In another mGWAS performed in 2018 by Rothschild et al. [100], 1046 healthy Israeli individuals who presented different ancestral origins and who shared a relatively common environment were analysed. This study, which could not be replicated, did not find any significant association between host genetic variations and bacterial taxa, but in conclusion suggested that some bacterial taxa were heritable [89]. 

In one of the most recent mGWAS published in Nature Genetics in 2021 [88], 18,340 healthy individuals from 24 cohorts were analysed to explore the host genetics and gut microbiome composition. Despite the large sample size, only one SNP rs4988235 in the *LCT* locus which was correlated with the abundance of *Bifidobacterium* showed an association at the genome-wide significance threshold (*p* = 1.28 × 10^−20^); this effect was the strongest in the Hispanic/Latin American cohort. Thirty-one taxon-specific microbiome trait loci (mbTLs), both for relative abundance or presence of microbial taxa, were identified as suggestive effects at a *p* < 5 × 10^−8^. Among them are the *FUT2* locus, which was associated with the abundance of *Ruminococcus torques* genus group, and three SNPs in the 9q21 locus which includes the gene *GCNT1*, encoding glycosyltransferase involved in the biosynthesis of mucin, and the *RFK* gene, which encodes the enzyme that catalyses the phosphorylation of riboflavin (vitamin B_2_). Even with the large sample size, the number of mbTLs identified was rather modest and the authors suggested that a larger sample size would have been necessary to increase the discovery rate. In this work [88], they also confirmed the likely influence of microbiome on the gut–brain axis and on gastrointestinal, brain and mood disorders. 

In the same year, a GWAS studying 8956 German individuals [101] identified 44 genome-wide significant associations with microbial composition in 38 genomic loci. Apart from the association of the *LCT* locus with *Bifidobacterium*, two independent univariate associations with a locus surrounding the histo-blood group ABO system transferase (*ABO*) gene were identified as associated with the differential abundance of *Faecalibacterium* (rs3758348, P _Meta_ = 6.16 × 10^−9^) and *Bacteroides* (rs8176632, P _Meta_ = 6.87 × 10^−10^*). Bacteroides* were also found to be significantly or suggestively associated with other variants in *BACH2* (P _Meta_ = 4.58 × 10^−10^) and *FUT2* (P _Meta_ = 4.46 × 10^−7^) genes. The authors then tried to infer the potential causal effect of microbiome on complex host traits by applying Mendelian Randomization (MR) analysis. MR analysis was performed for all univariate microbial features and 41 selected binary traits derived from the MR-Base database. None of the microbial traits with causal effects reached the genome-wide significance threshold at any locus, but some of them reached the suggestive threshold. It has been demonstrated that among the 19 suggestive (P < 1.22 × 10^−3^) microbial effects on host traits, nine of them were linked to IBD and Crohn’s disease. In particular, the presence of *Bacteroides* associated with ABO histo-blood group status appeared to significantly protect against Crohn’s disease development. Other MR results confirmed host–microbiome interactions previously described in observational studies. For example, from both in vivo studies and MR analysis, *Parabacteroides* were shown to have a protective effect on the “obesity class 2” trait [101].

To conclude, SNPs identified as having significant associations with microbial taxa did not overlap between the studies and replication of mGWAS results has been poor, apart from the association with *LCT* locus and the abundance of *Bifidobacterium*. This can possibly be explained by different statistical and technical methods, multiple-testing corrections and intrinsic sample features (lifestyle, diet, demographic and environmental conditions of subjects). However, the enrichment of microbiome-associated variants with immunity and metabolism related genes remains consistent among the studies. In addition, previous mGWAS have been conducted on a reduced number of individuals, mostly in Europe and North America. Future studies should consider larger cohorts in order to increase the power of these association studies, and including not only Western populations. In fact, the association of host genetic variants with microbiomes may be dependent on ethnicities, environment or intra-individual features, so studying diverse populations will help to identify these interactions. 

### 4.3. Evidence from Autoimmune Diseases and MS

As illustrated above, gut microbiome deeply influences the induction of immune responses. This influence and its interaction with host genetics could play a fundamental role in the development of autoimmunity, especially in those diseases in which genetic factors interact with environmental ones (smoking, diet, air pollution, hormone fluctuation, etc.) as risk factors, such as Inflammatory Bowel Disease (IBD), Rheumatoid Arthritis (RA), Systemic Lupus Erythematosus (SLE) and MS. These diseases share a common genetic architecture [30,102,103], increased pathogenic Th17 cells [104,105,106] and mechanisms related to gut dysbiosis [107,108,109]. As currently there are no studies correlating host genetic factors and microbiome in MS, in this review we describe this relationship in the abovementioned diseases, concluding with a brief report of a first study in EAE.

IBD is a chronic immune-mediated disease of the gastrointestinal tract which has two primary clinical manifestations: Crohn’s disease (CD) and ulcerative colitis (UC) [90,94]. IBD patients showed a decrease in the amount of *Firmicutes* and *Bacteroidetes* bacteria and an increase in *Proteobacteria* and *Actinobacteria* compared with healthy controls [94]. IBD susceptibility has a strong genetic component, with first-degree relatives (FDRs) of IBD patients having a higher risk of developing IBD [94]. Since the contribution of genetics and microbiome are clear, GWAS have been conducted to investigate the effect of host genetics on the gut microbiome in individuals diagnosed with IBD. These studies have led to the identification of more than 230 SNPs associated with IBD, even if they account for only a portion of heritability [94]. The majority of the IBD risk variants are located within immunity genes involved in interaction with the microbiome, confirming a role for host–gut microbes interplay in disease pathogenesis [110]. The first risk variant for IBD was mapped in the *NOD2* gene, which has the highest OR of 3.1 in Crohn’s disease. *NOD2* is expressed in gut epithelial cells, lymphocytes, monocytes and macrophages, acting as a defensive factor against intracellular bacteria and commensal microbes. It encodes an intracellular pattern recognition receptor that interacts with muramyl dipeptide (MDP), a peptidoglycan found in both Gram-positive and Gram-negative bacteria: the binding of MDP to *NOD2* induces activation of NK-B and MAPK and increases transcription of pro-inflammatory cytokines. *NOD2* deficiency results in a hyperinflammatory response to commensal microorganisms, while mutations in *NOD2* disrupt the delicate balance of immune homeostasis, which can lead to IBD [94,110]. In a mGWAS of 474 individuals, an enrichment of the family *Enterobacteriaceae* was found, especially *Escherichia coli*, in subjects with *NOD2* variants, supporting the idea that the proliferation of these bacteria is normally controlled by the *NOD2*-initiated immune response [90,94]. This result was confirmed by a second study conducted on 1514 healthy subjects in which *NOD2* was highly correlated with *Escherichia coli* abundance (4.6 × 10^−6^ < *p* < 1.3 × 10^−4^) [94]. Autophagy has also been involved in IBD: *ATG16L1, LRRK2* and *IRGM*, genes involved in the autophagic pathway, have been identified as risk-associated loci for this disease, especially CD [94,111]. Furthermore, two case-control studies confirmed the significant association between *ATG16L1* risk allele T300A and changes in gut microbiome architecture [111,112,113,114]. It remains unclear what direct role autophagy plays in IBD pathogenesis. Other GWAS studies identified *FUT2* as a risk locus for multiple diseases, including IBD and type 1 diabetes mellitus [90]. *FUT2*, which encodes a protein that transfers terminal fucose residues to mucin glycans, is involved in barrier function and is responsible for secretion of the ABO histo-blood group antigens in the mucosa [94,101]. Individuals carrying minor allele (A) lack terminal fucose residues on mucin glycans and are called “non-secretors”; non-secretors, who are homozygous for the loss of function nonsense mutation of the *FUT2* gene, have shown increased susceptibility to CD (OR 1.1, 95% CI, 1.071–1.143; *p* < 10^−15^). Moreover, *FUT2* non-secretors show a considerable decrease in the enrichment of *Faecalibacterium*, an anti-inflammatory and butyrate-producing genus, and an increase in *Proteobacteria*, demonstrating that *FUT2* polymorphisms are associated with microbiome [90,94,111]. Recently, in a cohort of 33 healthy individuals it was possible to replicate these results [94,115], even if they did not find a confirmation in a larger study of 1503 healthy twins [94,116]. 

RA is a multisystemic chronic autoimmune disorder that can affect the joints [117]. So far, studies at a genome-wide level have identified only 349 genetic variants associated with RA, explaining a small portion of heritability and disease pathogenesis. In parallel, it has been demonstrated that the composition of gut microbiome is different among patients, is heritable in 30% of patients and may be implicated in the pathogenesis as well. Therefore, genetic variants that cause a predisposition to RA may also be responsible for the interactions between microbiome, which explains part of the missing heritability, and RA pathogenesis. These risk loci are associated with immune function, as they mediate the transition to a pro-inflammatory phenotype, and they may involve the gut microbiome in this switch [117]. Dysbiosis has already been observed between RA patients and matching healthy controls, with *Prevotella copri (P. copri)* being the most variable bacterium in the two conditions (results confirmed in four of seven studies). Moreover, patients with an early disease onset showed immune activation against a *P. copri* peptide, Pc-27: in these patients, IgA and IgG responses were registered compared to controls. It is still to be clarified if the *Prevotella*–RA association is due to the host inflammatory condition or if it is causal [117]. In addition, transfer of fecal microbiota from mice susceptible to arthritis to germ-free mice led to an increase in susceptibility to the disease [118,119]. 

SLE is an autoimmune disease in which the immune system attacks its own tissues (joints, skin, brain, lungs, kidneys, and blood vessels), causing widespread inflammation and tissue damage in the affected organs. For what concerns the genetic component of the disease, about 100 risk loci have been identified, mostly in European and Asian populations [120]. Even if there are few studies on the association between microbiome and SLE, dysbiosis in patients has been observed. In particular, four independent studies (from Spain, China, and Netherlands) [109,121,122,123], with varying cultural, dietary, and geographic data, reported in patients a decrease in *Firmicutes* bacteria and an abundance of *Bacteroidetes* compared to healthy controls [118]. Another recent study found seven increased bacterial species and 19 decreased ones in SLE subjects, but none matched with the taxa previously identified in the aforementioned Chinese study [122]. An important study conducted on mice revealed *Enterococcus gallinarum* to promote a lupus-like disease [124]. *E. gallinarum* is a Gram-positive gut commensal bacterium of the Firmicutes which causes production of autoantigens, type I IFN and pro-inflammatory cytokines. Obviously, other studies are required to understand if the discrepancy between *Firmicutes* and *Bacteroidetes* is causal of the disease or is a consequence of it, and if this can affect the pathogenicity of other bacteria such as *E. gallinarum* [118]. 

The role of host genetics has been investigated in several animal models due to better control of genetics and environmental factors. Mouse models are considered a powerful model for evaluating host–microbiota interactions applicable to humans for two main reasons: high genetic similarity and similarity to humans at the microbial taxonomic level. For example, genetic background (i.e., BALB/c or C57BL/6J) has shown to be a stronger determinant than gender on the mouse microbiome [125], and the relative abundance of several heritable microbial taxa such as *Lactobacillus johnsonii* was identified as being affected predominantly by host genetics [126]. In the context of MS, a first study assessing the role of gut microbiome and host genetic interaction was recently performed on EAE [127]. Montgomery et al. demonstrated potent effects of host genetics on both EAE susceptibility and gut microbial composition predating disease onset. By using a genetically diverse mouse model representing 29 unique host genotypes, they studied EAE by microbiome sequencing and targeted microbiome manipulation. They identified specific gut bacteria and their metabolic functions associated with lower or higher EAE susceptibility across multiple host genotypes, implicating short-chain fatty acid metabolism as a key element conserved across multiple host genotypes: *Lactobacillus reuteri* was found as a commensal species capable of exacerbating EAE [127]. These results demonstrate the existence of complex interactions between host genetics and gut microbiota that modulate susceptibility to CNS autoimmune disease, providing insights into microbiome-directed strategies aimed at lowering the risk for autoimmune disease.

## 5. Conclusions

Research in the past decade has illustrated a close relationship between a host’s microbiome and health, which leads to questions about how these microbiotas form and whether host genes play a role. To date, the impact of host genetics on the composition of the gut microbiome remains unclear. Several studies in mouse models and humans support the idea that host genetic components can regulate gut microbiome composition: twin studies have shown that overall microbiome composition and many individual taxa are heritable. However, other studies in humans provide evidence for the dominant role of the environment in shaping the gut microbiome, while host genetics plays a negligible role. Although much effort has been invested, a clear trend indicating a role of the host genetics in the gut microbiome has not been identified, probably due to inconsistencies and unevenness between the studies’ results, low sample sizes and environmental factors [128].

Despite relatively strong epidemiological associations, the role of the microbiome in MS remains complex and unclear. It is now widely accepted that the gut microbiota plays a crucial role in the gut–brain axis, thus controlling CNS diseases, and dysbiosis is present in MS patients; however, we are very far from the identification of a particular gut microbiome signature in MS. This is mainly due to differences in geographical cohorts, sequencing techniques, stage of disease and treatment across the studies. However, the gut microbiome has emerged as a new environmental factor that could contribute to disease risk. At the same time, genetic architecture is also known to be involved in disease susceptibility; therefore, there is a strong need to understand how and if host genetics influence gut microbiome composition. The elucidation of this interaction could help in explaining the missing heritability determined by the interactions between environmental factors and genes. Studies in this direction are in their infancy, but a collective effort in this direction would be desired, addressing critical challenges such as increased sample size, independent replication, meta-analysis in multiple populations, and environmental factors. Overall, the comprehension of host genetics and gut microbiome composition at the onset of the disease could be a challenge for the design of personalized therapeutic strategies.

## Figures and Tables

**Table 1:** Gut microbiome studies in MS.

Observation/Microorganism	Host Organisms	Possible MS-Related Immunopathogenetic Mechanism	Reference
Germ-free gut	EAE mice	Milder disease course; lower levels of IFN-γ and IL-17A in the gut and CNS; Treg expanded in the CNS; disrupted BBB tight junctions.	[72] [73] [74]
Segmented Filamentous bacteria (SFB)	EAE mice	Higher levels of IL-17 in the gut; Th17 expanded in the CNS	[73]
*Bacteroides fragilis*	EAE mice	Polysaccharide A-mediated Tregs induction via TLR2 and suppression of the Th17 response	[76,77]
Decreased abundance of *Clostridia* [79], *Bacteroides* and *Parabacteroides* [79,80,81], *Butyricimonas* [82], *Faecalibacterium* [83], *Prevotella* [79,80,81], *Lactobacillus* [81], *Adlercreutzia* and *Collinsella* [81] with respect to controls	MS patients	SCFA-producer bacteria induce IL-10 dependent enhanced conversion of Treg cells and may be related to MS pathogenesis and progression; *Clostridia* can induce colon regulatory T cells (Tregs) and prevent autoimmunity; Epsilon toxin (ETX) produced by *Clostridium perfringens* has tropism for the blood-brain barrier (BBB) and oligodendrocytes/myelin.	[24,25] [85] [79] [86]
Increased abundance of *Metanobrevibacter* [82], *Acinetobacter calcoaceticus* [80,84], *Akkermansia muciniphila* [80,82,84], *Pseudomonas*, *Mycoplana*, *Blautia* and *Dorea* [81] with respect to controls	MS patients	Methanogenic archaea can induce DCs activation; *Akkermansia* and *Acinetobacter* have been associated with lower Treg induction and increased Th1 polarization. MS fecal transplant induced higher incidence of spontaneous EAE.	[24] [80,82,84]

**Table 2:** Microbiome GWAS studies.

Study	Year	Sequencing Method	Sample Size	Notes	Reference
Blekhman et al.	2015	Shotgun metagenomic	n = 93	Variants in the LCT gene correlated with abundance of *Bifidobacterium* (*p* = 1.16 × 10^−5^).	[95]
Davenport et al.	2015	16S	n = 127	SNPs in regions of the PLD1 gene associated with abundance of genus *Akkernabsia.*	[96]
Wang et al.	2016	16S	n = 182	Forty-two loci included variants in VDR gene-encoding (vitamin D receptor) associated with beta diversity (*p* < 5 × 10^−8^).	[97]
Turpin et al.	2016	16S	n = 1098 (discovery cohort) n = 463 (replication cohort)	Identification of 20 possibly heritable taxa.	[85,86,93]
Bonder et al.	2016	Shotgun Metagenomic	n = 1514	Confirmation of variants in the LCT gene correlated with abundance of *Bifidobacterium*. Nine new human loci associated with bacterial taxa and 33 loci associated with bacterial pathways.	[98]
Rothschild et al.	2018	Shotgun Metagenomic and 16S	n = 1046	No significant association detected.	[100]
Kurilshikov et al.	2021	16S	n = 18340	One SNP in the *LCT* locus correlated with abundance of *Bifidobacterium* (*p* = 1.28 × 10^−20^). *FUT2* locus was suggestively associated with the abundance of *Ruminococcus torques*.	[88]
Rühlemann et al.	2021	16S	n = 8956	Thirty-eight genetic loci found to be associated with single bacteria and overall microbiome composition. ABO group was suggestively associated with the abundance of *Faecalibacterium* (P _Meta_ = 6.16 × 10^−9^) and *Bacteroides* (P _Meta_ = 3.65 × 10^−10^).	[101]
